# Design of Surface Enhanced Raman Scattering (SERS) Nanosensor Array

**DOI:** 10.3390/s20185123

**Published:** 2020-09-08

**Authors:** Yaakov Mandelbaum, Raz Mottes, Zeev Zalevsky, David Zitoun, Avi Karsenty

**Affiliations:** 1 Advanced Laboratory of Electro-Optics (ALEO), Department of Applied Physics/Electro-Optics Engineering, Lev Academic Center, 9116001 Jerusalem, Israel; ymandelb@g.jct.ac.il (Y.M.); rm1995ex@gmail.com (R.M.); 2 Faculty of Engineering, Bar-Ilan University, 5290002 Ramat Gan, Israel; Zeev.Zalevsky@biu.ac.il; 3 The Nanotechnology Center, Bar-Ilan University, 5290002 Ramat Gan, Israel; David.Zitoun@biu.ac.il; 4 Faculty of Exact Sciences, Department of Chemistry, Bar-Ilan University, 5290002 Ramat Gan, Israel; 5 Nanotechnology Center for Education and Research, Lev Academic Center, 9116001 Jerusalem, Israel

**Keywords:** optical nanosensor, plasmon, modeling, simulations, real-time detection

## Abstract

An advanced Surface-Enhanced Raman Scattering (SERS) Nanosensor Array, dedicated to serve in the future as a pH imager for the real-time detection of chemical reaction, is presented. The full flow of elementary steps—architecture, design, simulations, fabrication, and preliminary experimental results of structural characterization (Focused Ion Beam (FIB), TEM and SEM)—show an advanced SERS pixel array that is capable of providing spatially resolved measurements of chemical pH in a fluid target that became more than desirable in this period. Ultimately, the goal will be to provide real-time monitoring of a chemical reaction. The pixels consist of a nanostructured substrate composed of an array of projections or cavities. The shape of the nanostructures and the thickness of the metallic (Ag or Au) layer can be tuned to give maximal enhancement at the desired wavelength. The number and arrangement of nanostructures is optimized to obtain maximal responsivity.

## 1. Introduction

A new frontier in chemical sensing is the development of real-time sensors that are capable of monitoring continuous flow reactions. To this end, an imaging sensor that can record and report spatial variations in real time is desired. Surface-Enhanced Raman Scattering (SERS) is capable of chemical sensing yet currently is performed either on chemicals that are adsorbed on a particular substrate, by scanning with a sharp metallic tip [1,2], or by dispersing metallic nanoparticles into the solution. The ability to analyze the composition of a mixture on the nanoscale makes the use of SERS substrates beneficial for environmental analysis, pharmaceuticals, material sciences, art and archeological research, forensic science, drug and explosives detection, food quality analysis [3], and single-algal cell detection [4,5,6]. SERS combined with plasmonic sensing can be used for a high-sensitivity and quantitative detection of bio-molecular interaction [7] and to study redox processes at the single molecule level [8]. 

### 1.1. The Need for Real-Time Monitoring of a Chemical Reaction 

The architecture, design, simulation, and fabrication of an advanced SERS pixel array to provide a spatially resolved measurement of chemical pH in a fluid became more than desirable. Ultimately, the goal is to provide real-time monitoring of a chemical reaction. The pixels consist of a nanostructured substrate composed of an array of projections or cavities. The shape of the nanostructures and the thickness of the metallic (Ag or Au) layer can be tuned to give maximal enhancement at the desired wavelength. The number and arrangement of nanostructures is optimized to obtain maximal responsivity. 

### 1.2. pH Determination and Raman Spectroscopy

Initial designs for Raman and pH measurement endeavored to enhance the Raman signal of the analyte; by gauging the strength of the signal, one may determine the concentration. In particular, pH is the (base 10) logarithm of the $H^{+}$ concentration, $\mathrm{pH}\;=\;-\log_{10}\left(\left[H^{+}\right]/\left[H_{2}O\right]\right);$ determination of the free proton concentration [$H^{+}$] (or the hydronium concentration [$H_{3}0^{+}$]) gives the pH. Since water can be said to dissociate into hydronium and hydroxide ions —$2\;H_{2}0\;↔{\;H}_{3}0^{+}+{\;\mathrm{OH}}^{-}$—by the law of mass action, the product of the (relative) concentrations of hydrogen and hydroxide ions is essentially constant at any given temperature, [$H^{+}$] [OH] = *C*(*T*); at room temperature, the product is 10^−14^. Hence, detection of the ${\mathrm{OH}}^{-}$ Raman signal will do just as well as $H^{+}$ ($H_{3}0^{+}$). This approach suffers from several drawbacks: first, the signal weakness, since ${\mathrm{OH}}^{-}$, $H^{+}/H_{3}O^{+}\;$have exceedingly small Raman cross-sections [9]. Second, the uncertainty in distance to surface. Unless significant measures are taken to maintain a pure environment, metal surfaces will invariably adsorb various molecules creating a layer up to 1–2 nm thick. Thus, the analyte’s molecules are prevented from adsorbing directly onto the nanostructure surface. The separation may seem minor, but near the surface, the electromagnetic enhancement factor falls off very steeply. Hence, the uncertainty in the distance from the surface makes it very difficult to accurately predict the enhancement factor experienced by the analyte [10].

One solution to the aforementioned problem is to react the analyte directly onto the metal surface. However, noble metals do not react in most of the pH range, and non-noble metals are corroded and undergo competitive reactions with the counter ions; desorption is nontrivial and essentially every value of the pH to be measured requires the fabrication of a disposable device. Real-time dynamic measurements are clearly precluded.

In order to avoid these problems, in recent years, a different design for SERS pH measurement was developed, in which the pH is determined indirectly. A metal-bonding molecule, typically a thiol such as (para-) mercaptobenzoic acid (pMBA) is adsorbed directly onto the surface. These are the molecules that experience the field enhancements and whose Raman spectra are recorded. The thiols bind on one side to the metal, Ag or Au. On the other side of the benzyl ring, a carboxyl group is attached. The vibrational dynamics of the carboxyl group O=C-OH are dominated by the stretching mode of the double bond C=O around 1700 cm^−1^. At high pH levels—basic solutions—the conjugate base is preponderant with a carboxylate group COO– with a vibration around 1400 cm^−1^. The ratio of the two peaks can be taken as a calibration curve for the quantitative monitoring of the pH [11].

Then, in this set-up, the pMBA molecules become an additional, permanent component of the device. One might consider the device to be the complex comprised of the metal nanostructures together with the attached thiol molecules. They experience the full enhancement as they are directly adjacent to surface. Conversely, the fact that the analyte does not bond directly to the surface opens the possibility of reusability. This approach has been successfully applied to Au colloids, specifically to Au nanospheres to increase the surface area [11,12]. In both cases, the plasmonic effect was observed on the rough surfaces of the nanoshells without a precise knowledge on the hotspots. Turnover times of approximately 10 min, for rinsing in a buffer solution, are reported by Bishnoi et al. [11] and Talley et al. [12], who simply state that “buffer solution was flowed through the flow cell” between pH increments. Thus, relatively rapid recycling times are attainable, and conceivably dynamic measurements are possible as well. The colloidal approach has been applied to image the pH in living cells [13]. The literature has been summarized in reviews [10,14], while more recent work has been achieved on gold colloids to increase the number of hot spots in hyperbranched Au [15]. On the other hand, the plasmonic nanostructures have also been obtained from nanofabrication as an array of nanoneedles [16] on a polymeric substrate, which demonstrated pH sensing on a mechanically stable array. In this pioneering work, the influence of the morphology of the needles on the SERS enhancement has not been discussed and has motivated the present work.

Following excitation using a focused laser beam, the design by which the Raman-scattered radiation will be collected must be considered. In the preliminary stage, this will be accomplished using one of the existing methods, using lens-based imaging. In general, Raman imaging is nontrivial, since it amounts to hyperspectral imaging combining spatial location with frequency. The ‘classical’ methods include point scanning, line scanning, and wide field mapping. All three involve mechanical scanning, whether directly in the spatial domain, for the first two—with output fed into a spectrometer—or in the spectral domain for the third. In the last case, spectral scanning can be accomplished directly as by a grating and monochromator, or by the Fourier Transform method (wherein a wideband signal is fed into the arm of a Michelson–Morley interferometer, and one arm is scanned, thus varying the phase difference). These are inherently slower and require alignment. Confocal illumination is also typically required to remove out-of-focus emission. Polarization of the excitation and filtering of the collected light by a Phosphate-Buffered Saline (PBS) is also used to improve contrast [2]. However, due to high axial (depth) localization of the enhancement near the nanostructures and the low Raman response of the analyte (H+ and OH-) itself, one expects such measures to prove unnecessary. Newer methods that give instantaneous hyperspectral imaging include Fiber Array Spectral Translation (FAST) and Liquid Crystal Tunable Filter (LCTF) [9]. Thus, the latter are to be preferred. In particular, in a FAST-based design, the emitted Raman radiation is imaged by an optical objective onto an array of optic fibers (FAST), each pixel onto one fiber (at least); these emit the collected light through a dispersive element (such as a grating), separating it into its component spectra, onto an array of photodiodes, gated detectors or onto a Charge-Coupled Device (CCD), one row per fiber. Thus, the Raman spectrum of each pixel is imaged simultaneously. In later stages of the first-generation device, the aim is to replace the lens-based imaging—with its inherently larger distances—with a direct collection by a proximally located Fiber Array. Communication lines will include two arrays of waveguides for delivery and collection of the light to each one of the pixels in the array. The challenge will be to design a grid of baffles to prevent *crosstalk*—the unwanted collection of radiation from neighboring pixels. Plasmonic nanoantennae coupled to plasmonic waveguides for collection and excitation will be considered as a more compact alternative for second-generation design.

## 2. Principles and Formalism

**Figure 1:**
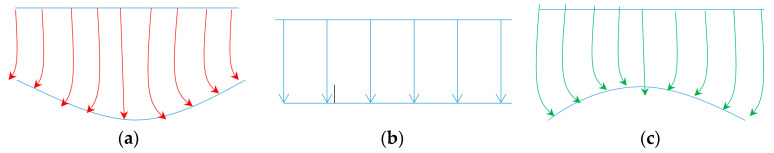
Schematics of the Lightning Rod Effect: Field lines must be normal to a conductor. Thus, the conservation of flux requires that the field lines bunch together—i.e., the field increases—when impinging on a protrusion; they must spread apart—the field decreases—on the inside of a depression. (**a**) Diverging lines; (**b**) Parallel lines; (**c**) Converging lines.

In order to explain the way the device acts as an optical array of nanosensors, we may first introduce some concerns and considerations in combining several physical effects and configurations together; then, we present the formalism of its functionality. The concepts of Raman effect, surface plasmon polaritons, and localized surface plasmons are detailed in the supporting information. The lightning rod effect is often described as a non-resonant effect by which electric fields are intensified in the vicinity of metal surfaces of high positive curvature such as a tip or a corner. Conversely, the fields are suppressed near metal regions of high negative curvature as at the apex of a tip-like cavity or near an internal corner. For a metal, the explanation can be followed on: the field lines must be orthogonal to the surface (Figure 1). A surface with sides bent backward, creating a protrusion, concentrates the field lines causing the field strength to increase; if the sides are bent forward, forming a depression, the field lines diverge, and the field is reduced.

The Surface-Enhanced Raman Scattering (SERS) occurs due to the coupling of two phenomena: (1) (Spontaneous) Raman Scattering—vibrational/phonon modes mix with an optical excitation, leading to radiation with a frequency shift, and (2) Surface Enhancement—Localized Surface Plasmons (LSP) and/or Propagating Surface Plasmons (PSPP) lead to enhancement of the excitation and of the scattering fields.

**Figure 2:**
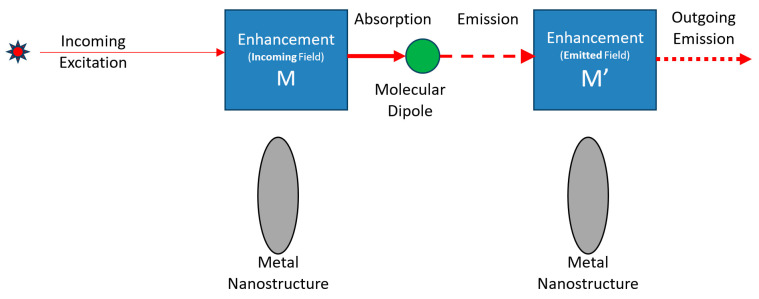
Schematics illustration of a Surface-Enhanced Raman Scattering (SERS) process flow: Incoming excitation is enhanced by a factor of M, absorbed and reemitted by the molecular dipole, and then enhanced by a factor of M’. Thus, the total enhancement is M × M’.

In spontaneous Raman scattering, incident radiation is absorbed by a molecule and reradiated at a shifted frequency. The intensity, *I*_em_, of the emitted radiation is linear with respect to the intensity *I*_inc_ of the incoming exciting radiation field in the vicinity of the molecule. In the presence of a metallic structure, the field intensity of the local excitation is enhanced by a factor, *M*, which is known as the “Local Field Intensity Enhancement Factor” (LFIEF). This can reach in excess of 10^5^. Thus, the radiation emitted by the molecule is enhanced by the same factor. However, upon emission, the metal structure causes enhancement of the *emitted* radiation, by essentially the same factor *M*, according to the Optical Reciprocity Theorem (ORT). Hence, the Raman-shifted radiation that is detected in the far field is enhanced overall by a factor of *M*^2^, which can be in excess of 10^11^ [1]. The process is portrayed schematically in Figure 2. More details are available in the supporting information.

Two examples, which will prove important in the following, are an ellipsoid of revolution and a cone. If an ellipse is rotated about one of its axes, one obtains a surface of revolution also known as a *spheroid*. If the major axis is chosen, one obtains a *prolate* spheroid; the curvature at the north pole (N) is greater than at the equator or meridian—the object is ‘pointier’ there than a sphere. If a metallic prolate spheroid is placed in an electric field oriented along its symmetry axis, the LFIEF at (N) is increased relative to that of a sphere by a factor $\approx {\left(\frac{1}{2}\left(\frac{1}{L_{3}}\;-\;1\right)\right)}^{2}$ where *L_3_*, known as a depolarization factor, is a function of the eccentricity; it is equal to 1/3 for a sphere and *L_3_* < 1/3 for a prolate spheroid. The higher the eccentricity, the smaller is $L_{3}$ and the more the LFIEF is augmented. The SERS EF is increased similarly by a factor $\approx {\left(\frac{1}{2}\left(\frac{1}{L_{3}}\;-1\right)\right)}^{4}$ [1,17].

The electric field near the apex of an ideal cone can be shown [18] to vary with the distance *r* as

(1) $$E\sim r^{\nu -1}.\;$$

For a cone, the exponent is ν < 1; the field diverges near the tip. If the cone angle increases past 90°, the cone becomes a cavity. In this case, the exponent increases to 1 < ν, so the field near the point vanishes.

### 2.1. Case of the Ellipsoid

**Figure 3:**
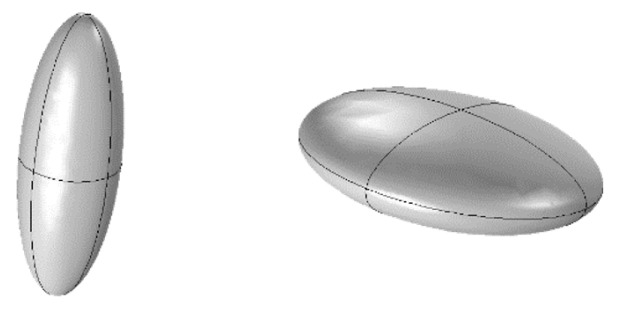
Spheroids. (**Left**) Prolate spheroid; (**Right**) Oblate spheroid.

If a sphere is deformed into a prolate spheroid, an egg-like shape (Figure 3), with eccentricity *e*, then the field enhancement for a field polarized along the direction of the major axis, the ‘pointy’ axis, is increased. For an oblate spheroid, a squashed patty, the field enhancement along the ‘squashed’ axis is reduced.

Using ellipsoidal coordinates in the Electrostatic Approximation (ESA) [1,19], one finds that the maximum LFIEF occurs at the north pole on the *outer* boundary. The minimum LFIEF occurs on the meridian where the field is tangent to the surface and hence equal to the field inside, which is uniform: *M*_min_ *= M*_in_. The maximum and minimum LFIEF are listed in Table 1.

Here, $\epsilon_{M}$ is the dielectric constant of the surrounding medium, and *L*_3_ is a geometrical factor called the “depolarization factor”. For a sphere, *L*_sphere_ = 1/3; for a prolate spheroid, *L* < 1/3; for an oblate spheroid, *L* > 1/3. The resonance condition follows from the denominator of (2).

This reduces to $\epsilon_{\mathrm{re}}\left(\lambda_{*}\right)\;=\;-2\epsilon_{M}$, for a sphere where *L_3_* = 1/3. For typical metals, for a prolate spheroid, the resonance is red-shifted to longer wavelengths relative to the sphere, while for an oblate spheroid, it is blue-shifted to shorter wavelengths. Referring to (7) for a prolate spheroid, the enhancement is increased relative to the sphere, (5), while for an oblate spheroid it is reduced by a factor $\left(1\;-\;L_{3}\right)/L_{3}{)}^{2}$; this is an expression of the Lightning Rod Effect [17].

An alternative configuration under consideration involves nanocavities rather than protrusions. It is important to compare the enhancement attainable for the two types of geometries, in order to choose the optimal geometry. In this context, a curious duality is noted between particles and cavities, which exchanges the roles of prolate and oblate spheroids; another duality is noted between the major and minor axes of any particular spheroid. This duality is significant in choosing the optimal shape for a given excitation polarization and vice versa. An analysis of a prolate spheroid cavity in the ESA is considered here. The results of the previous discussion can describe a cavity within a metal under the observation [19] that one need only switch the values of the dielectric functions between the medium and the nanostructure to describe a cavity. In addition, in the case of a cavity, it is the field *within* the boundary that is relevant. Thus, the relevant expression is (4), which describes the enhancement within the structure. Exchanging $\epsilon \left(\lambda \right)↔\epsilon_{M}$ gives the expressions listed in the table above for the enhancement, the resonant condition, and the value of the enhancement at resonance. For instance, the enhancement for a spherical cavity is reduced by a factor of 1/16 relative to a spherical particle; the SERS enhancement suppressed by a factor of 1/256 ≅ 0.004.

For a propagating field, the expressions above for the enhancement in the cavity must be multiplied by a correction factor ${\left|\epsilon_{M}/\epsilon \left(\lambda_{*}\right)\right|}^{2}$ to account for the reduction of the excitation field in the metal surrounding relative to free space. Thus, the true enhancement for the cavity is:

(9) $$M_{\mathrm{cav}}\;=\;{\left|\frac{\epsilon_{M}}{L_{3}\epsilon_{M}\;+\;\left(1\;-\;L_{3}\right)\epsilon \left(\lambda \right)}\right|}^{2}$$

which at resonance becomes:

(10) Mcav(λ*) = 1 (1 − L3)2|ϵMImϵ(λ*)|2

The enhancement for a spherical cavity is only reduced by a factor of 1/4 relative to a spherical particle, and the SERS EF is only reduced by a factor of 1/16. This has a significant caveat: the correction ${\left|\epsilon_{M}/\epsilon \left(\lambda_{*}\right)\right|}^{2}$ on resonance is greater than 1, which is not physical. In addition, depending on the metal, the reduction may be mitigated somewhat due to the change in resonance wavelength.

Regarding the duality, a very interesting relation may be discerned in expressions (3), (6), and (8) regarding the relative enhancement $\tilde{M}$ in the cavity; these are precisely the expressions for the enhancement and for the resonance conditions at the (outer) north pole of a nanoparticle, but with *L_3_* replaced by ${\tilde{L}}_{3}=\;1-L_{3}$. Thus, if the cavity in question is a prolate spheroid, it behaves similar to an *oblate* spheroid particle (and vice versa if 2/3 < *L_3_*). In particular, a prolate cavity will enhance a field polarized along its symmetry axis more *weakly* than a spherical particle, and the oblate cavity will enhance more strongly.

### 2.2. Nano-Cones

The non-resonant enhancement referred to as “The Lightning Rod Effect” can be investigated by itself without resonant excitation of an LSP using the geometry of a cone. A simple analysis was performed in [18] for a perfectly conducting material in an electrostatic field. Near the tip, the radial component of the electric field varies as

(11) $$E_{r}\;\sim \;r^{\nu \;-\;1}P_{\nu }\left(\cos\theta \right)$$

where $P_{\nu }\left(x\right)$ is a Legendre function of the first kind and order ν; ν may be non-integral [20]. It follows from that for ν < 1, the field will be enhanced near the tip, where r is small; for ν > 1, it will be suppressed.

**Figure 4:**
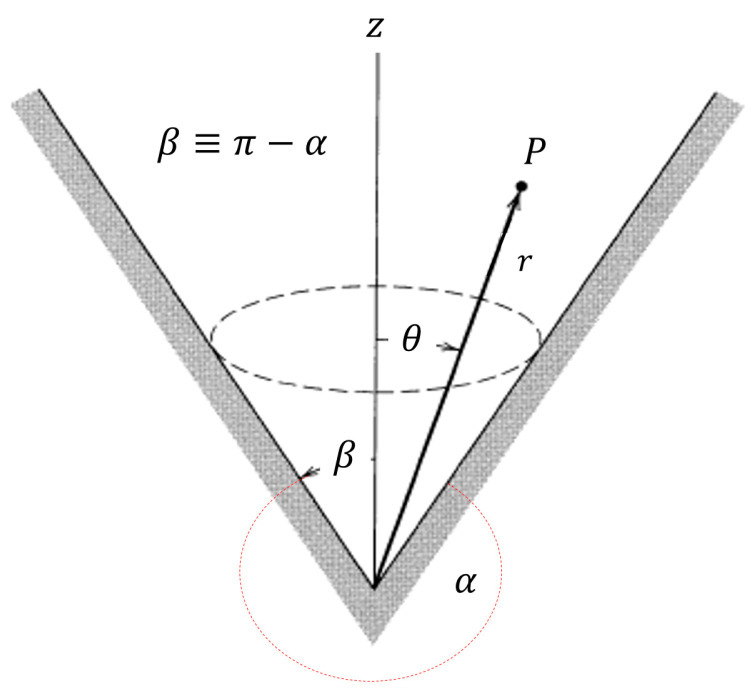
Dependence of ν on the cone semi-angle in a real metal. At high α (low β), corresponding to a cavity, 1 < ν. E is suppressed.

**Figure 5:**
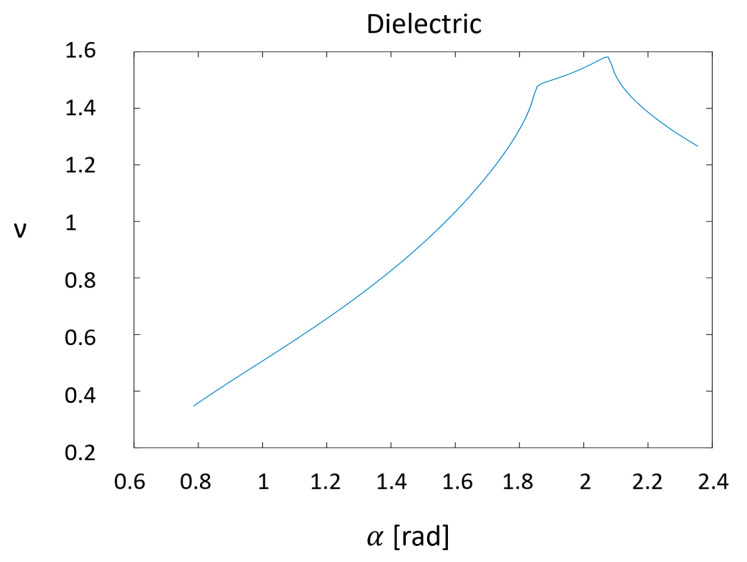
Graph of ν as a function of α.

An analysis for a propagating field and a real metal with a dielectric function with a negative real part and a nonzero imaginary part was carried out in [21]. However, the authors did not continue their investigation to values of the angle π/2 < α, which describes a cavity. This analysis has been continued here using Matlab. It is clear from Figure 4 and Figure 5, as in the case of the perfect conductor, that for π/2 < α, the exponent ν < 1, implying that the field near the apex of a conical cavity is suppressed. (For large values of α, which describe a narrow cavity, the results are erratic; this is presumably due to round-off error). The dependence of ν on the cone semi-angle in a real metal is presented in Figure 4, and the graph of ν as a function of α is presented in Figure 5.

### 2.3. Nanoshells

The enhancement for a dielectric (or real metal) shell is dependent on the internal and external radii. The field is a ‘hybrid excitation’ of the internal and external surfaces. The dependence is such that adjusting the internal and external radii allows one to tune the location of the resonance peak; in particular, it may be chosen to match the wavelength of the excitation source available in the laboratory and still use a low-absorption material [22,23]. Stratified ellipsoids and other shapes can also be created, and the extra enhancement they provide may be tuned as well [24,25]. The sphere has external radius *b*, internal radius *a*, and hence thickness *b* – *a*. In the ESA, the field enhancement at the North pole (N) [26] is:

(12) $$M\;=\;{\left|\frac{\epsilon \epsilon_{M}\;+\;\frac{2}{3}\left(\epsilon \;-\;\epsilon_{M}\right)\epsilon \Delta }{\epsilon \epsilon_{M}\;+\;2{\left(\frac{\epsilon \;-\;\epsilon_{M}}{3}\right)}^{2}\Delta }\right|}^{2}$$

where:

(13) $$\Delta \;\equiv \;\left(1\;-\;\frac{a^{3}}{b^{3}}\right)$$

The solid sphere corresponds to Δ = 1, while the shell of vanishing thickness is described by Δ = 0. In the latter case M → 1, as consistency demands.

Resonance occurs when (12) is maximal. For a solid structure, the resonance is achieved for a particular wavelength, which is determined by the form of ϵ(λ). By contrast, expression (12) can be maximized for any value of λ by setting Δ appropriately. Thus, one may choose a convenient wavelength and achieve resonance by tuning the thickness of the shell.

**Figure 6:**
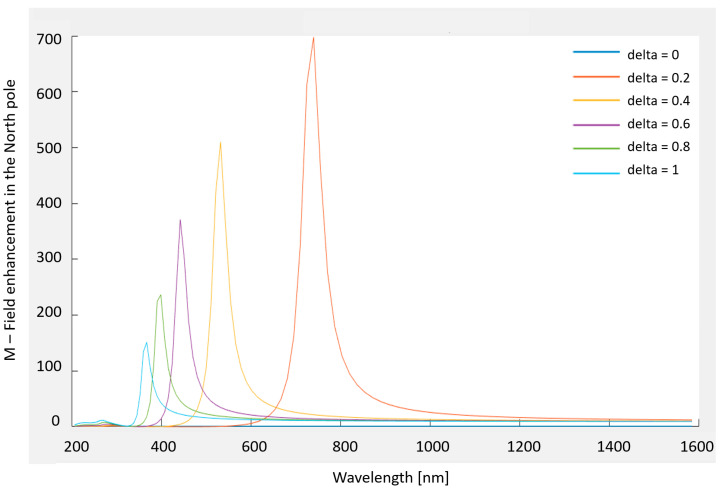
Matlab plot of the field enhancement at the north (N) pole.

A Matlab code was used to plot (Figure 6) the field enhancement at the north (N) pole.

### 2.4. Multiple Nanostructures

#### 2.4.1. Multiple Nanostructures Influence

**Figure 7:**
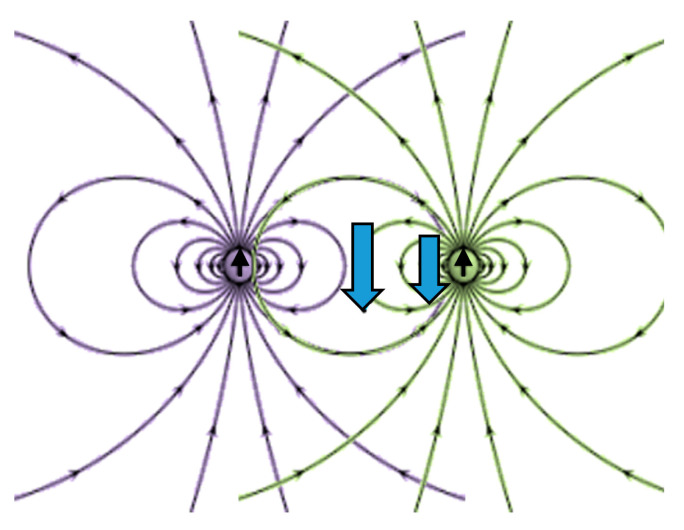
Neighboring static dipole—mutual suppression.

Nanostructures that are in proximity have the potential to influence one another. For instance, a uniform electric field induces a dipole moment in a nanoparticle parallel to the field. The (external) electric field generated by the dipole moment is parallel to the initial field along the axis parallel to the field, and it is diametrically opposed in the equatorial plane orthogonal to this axis. The field of a dipole is illustrated in Figure 7. Neighboring particles situated sufficiently nearby along the axis will experience augmented enhancement, while the enhancement of nearby particles located in the equatorial plane will be reduced. Thus, in the electrostatic approximation—placing the two structures in a constant uniform field so that the axis between them is orthogonal to the field—one expects the two structures to suppress one another. 

**Figure 8:**
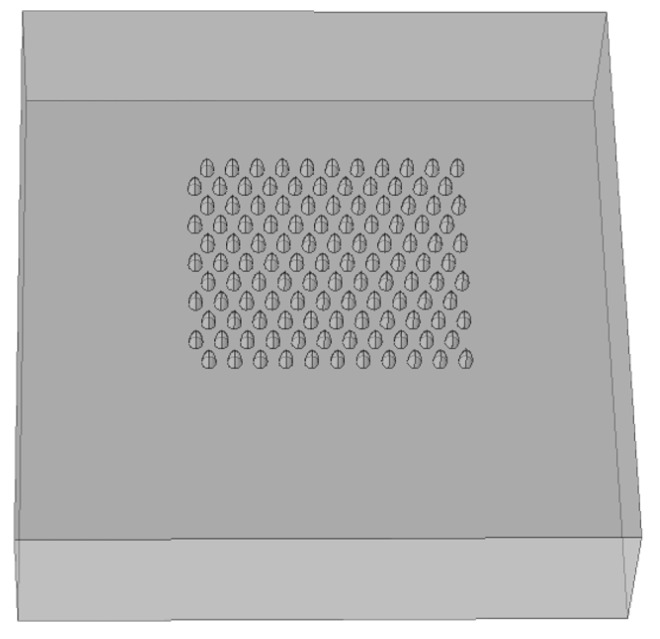
Comsol rendering of a pixel based on a hexagonal lattice.

For cavities, the induced dipole is *less* than would be there without the cavity; the total induced dipole is antiparallel to the field. Thus, the situation is reversed: on the axis, the external field is reduced; in the equatorial plane, it is *augmented*. The Comsol rendering of a pixel is presented in Figure 8. 

An alternate description contends that nanoparticles in close proximity allow the excitation of gap or hybrid modes [1]. When the field is aligned with axis of the gap, a symmetric hybrid mode is excited as a result and the field is increased; when the field is orthogonal to the axis, the antisymmetric mode is excited, and the field is reduced. It is of note that the interactions described are *non-radiative*.

#### 2.4.2. Total Raman Emission Cross-Section

In designing a SERS detector, ultimately, the responsivity depends on the total detected radiation and not just on the relative enhancement. For a nanometric structure, the total Raman intrinsic (non-SERS) scattering cross-section of the adjacent molecules may be very small, due to the small surface area. Even very strong enhancement may result in a signal too weak to be reliable. For colloidal nanoparticles dispersed in solution, the large number of particles finesses the problem. Metamaterials with numerous nanostructures assembled on a substrate have also been demonstrated to be effective [27]. For the device in question, this too is not an option; the demand for spatial specificity inherent in the notion of an imaging pixel limits the design at most to small collections of closely space structures well-separated from one another.

The responsivity, and hence the signal-to-noise ratio, can be greatly increased by using a pixel design based on a finite array of multiple structures. The spacing between the structures will serve as a parameter for optimization. As discussed, two neighboring structures subjected to a field orthogonal to the axis between them tend to suppress one another through non-radiative interactions. In an array, each structure has several neighbors and the suppression increases; it decreases with the distance. The more structures there are, the greater the total Raman scattering; however, if the pixel size is kept constant, more structures means smaller separation and hence greater suppression. An optimal separation (or equivalently an optimal number of structures) for which the total scattering is maximal is expected.

For the sphere, one can analytically estimate this optimal separation as a function of dielectric constant and the number of neighbors; the expression is simple, and MATLAB can be used. For a hexagonal lattice (6 neighbors) of silver nanospheres with an excitation wavelength of 500 nm, the optimal separation is about 60 nm. For the ellipsoid, the estimate involves (inverting) elliptical integrals, which must be evaluated numerically; the advantage over an all-numerical FEM calculation with COMSOL dwindles. 

## 3. SERS Biochemical Optical Nanosensors Array

### 3.1. On-the-Chip pH Raman Imager

**Figure 9:**
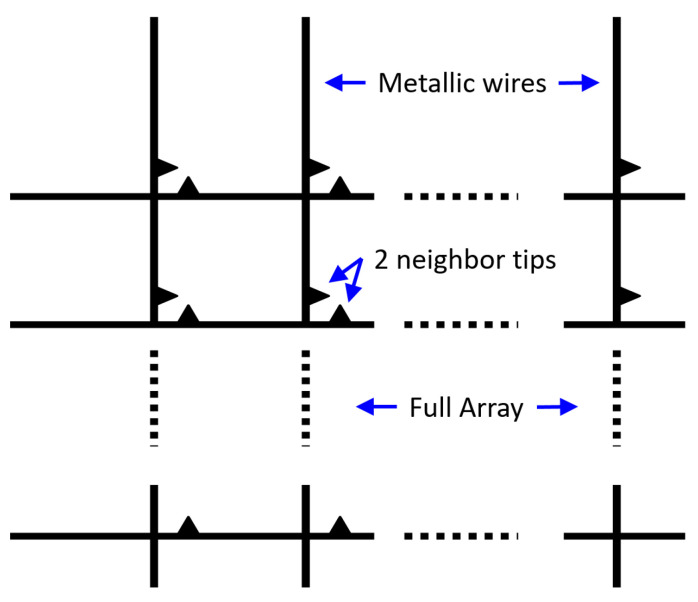
Schematic picture of the metallic wires with edges.

**Figure 10:**
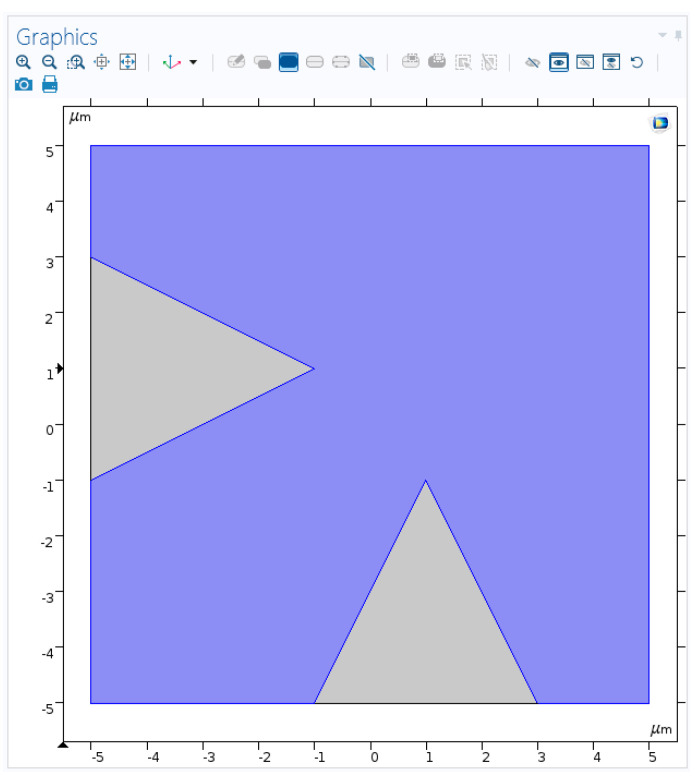
Comsol structure simulation of neighbor tips for enhancement.

**Figure 11:**
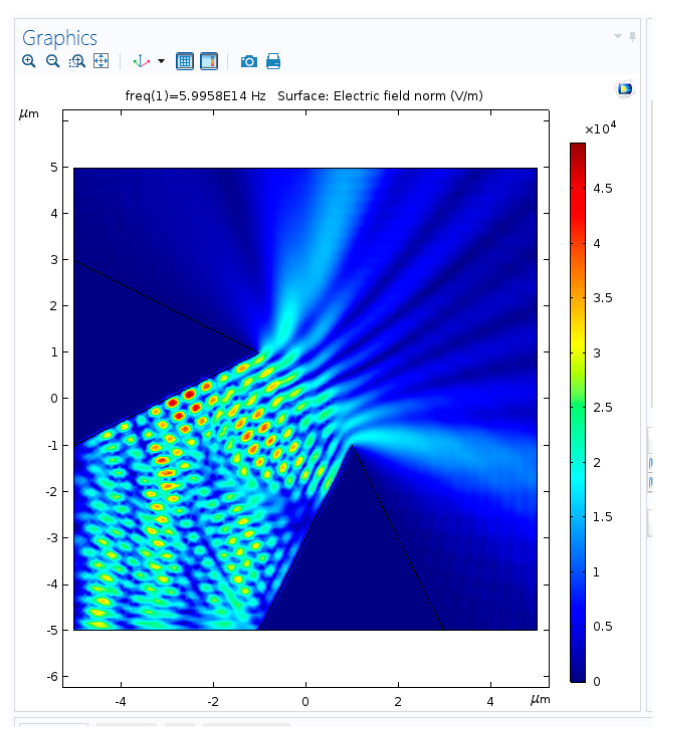
Comsol surface electric field simulation of neighbor tips for enhancement.

**Figure 12:**
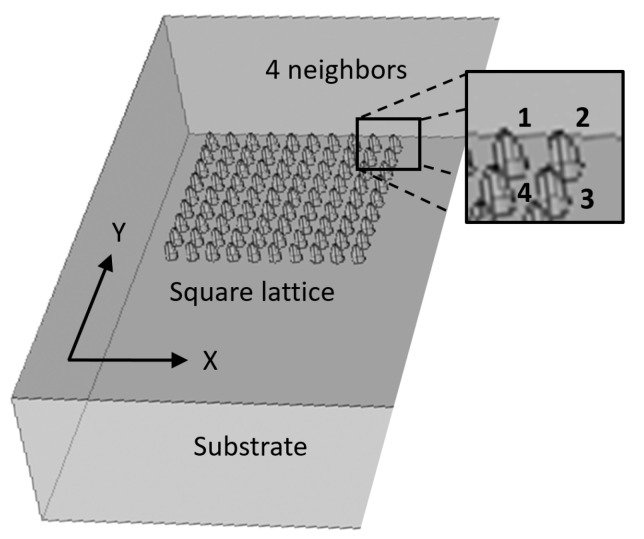
Nanocones design. Square lattice showing the four nearest neighbors.

**Figure 13:**
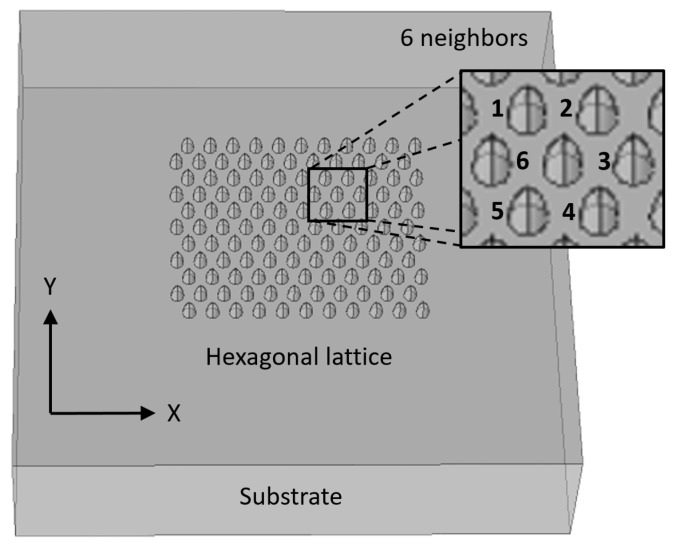
Nanocones design. Hexagonal lattice showing the six nearest neighbors.

**Figure 14:**
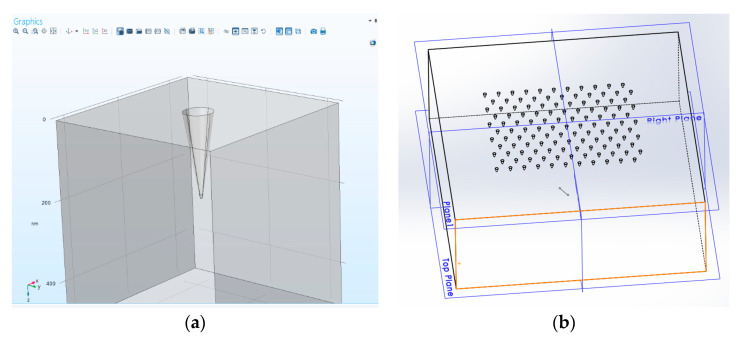
Nanoholes design. (**a**) Single cavity element. (**b**) Hexagonal lattice showing the six nearest neighbors. Holes are simpler to drill using Focused Ion Beam (FIB).

The idea is to generate an array of enhanced Raman scattering pixels to realize a Raman imager for the application in the construction of an on-line improved chemical sensor monitor of temporal and spatial dynamics of chemical process. During the design phase, four main configurations were considered in order to check what should be the optimal geometry for an array of pixels with enhancement phenomena: planar metallic wires with edges (Figure 9, Figure 10 and Figure 11), nanocones in square lattice (Figure 12), nanocones in hexagonal lattice (Figure 13), and nanocavities in hexagonal lattice (Figure 14).

A first proposal is to have a device constructed from an array of metallic wires with edges that will generate the Raman enhancement in every “pixel” or cell of interaction in which the chemical measurement will be performed. The array of metallic wires will look similar to what appears in Figure 9. Next to the array of metallic wires, there will be an array of light-guiding material that will deliver the excitation light to each one of the pixels in the array as well as collect the light scattered from each one of the pixels. The collected light will be guided to a separate analysis region in which a planar and compact spectrometer will be realized. The data after the spectroscopic analysis will be delivered to an electronic interface that will visualize the Raman “image”. The structure of the optical waveguides will look in a way similar to the structure of the metallic wires of Figure 1 but will be made from light-guiding materials rather than metals. One thing to be considered is to guide plasmonic modes to generate a much more spatially compact interaction between the light and the chemical material that we aim to measure in each of the pixels of our Raman imager. In this case of realization, the waveguide will be an interface between metals and dielectric materials, while the “lens” for illuminating each pixel (for excitation) and for collecting the light scattered from each pixel will be realized with plasmonic nanoantennas.

### 3.2. Enhancement Structures

Raman scattering is very weak. In Surface-Enhanced Raman Scattering, several mechanisms enhance both the incoming excitation and emitted radiation: the Localized Surface Plasmons (LSP)—i.e., a resonant plasmonic effect—and the Lightning Rod Effect (LRE)—i.e., electric fields intensify near metal points. Coupled-LSP resonances arising from the interaction of two closely spaced metallic objects—gap-Plasmon resonance—typically exhibit the largest local field enhancements, and accordingly, they play an important role in SERS. Such enhancement is demonstrated using Comsol software, while a two-neighbor tips structure (Figure 11) is supposed to create an increased surface field (Figure 12). In fact, there is a spacing parameter to consider. Given the micron spacing between the structures, no serious coupling is expected between the adjacent structures, and the distance between the adjacent edges should be decreased in order to obtain significant enhancement. Usually, the Surface Plasmon (SP) behaves as an antenna, and it can sense other plasmons until the distance in which the electrical field decreases in dielectric medium. The following parameters are defined:

- δ_diel_ is the spacing for which it is possible to sense other plasmons: approximately 250 to 1000 nm,
- δ_metal_ is the penetration depth of the electrical field inside the metal: approximately 5 nm,
- δ_SPP_ is the distance where the electrical field extends along the metal: approximately 2–20 µm for λ = 500 nm.

### 3.3. Square and Hexagonal Lattices

Additional configurations of nanocones were also studied in parallel during the design phase, such as a square lattice of pixels (Figure 12) and hexagonal lattice of pixels (Figure 13). The main difference between the two structures is the spacing between the adjacent tips. While in square configuration, rows and columns are reproduced without any shift, in hexagonal configuration, the rows are shifted and enable six adjacent tips, instead of four in square geometry. It appears that the number of neighbors is increasing from 2, in planar metallic lines configuration (Figure 9), to 4 in square lattice configuration (Figure 12), and to 6 in hexagonal configuration (Figure 13). Looking for enhancement phenomenon, it seems that the hexagonal solution is more adequate at this stage. The last improvement would be to choose a nanocavities hexagonal lattice (Figure 14), since it is much easier in Focused Ion Beam (FIB) fabrication.

## 4. Methods: Analytical, Numerical, and Experimental

### 4.1. Combining Analytical and Numerical

As presented in the Formalism section, several analytical models are desirable of course in order to define mathematically physical behaviors and case studies of integrated effects into a device or a module. Among other relevant analytical relevant methods in our SERS case study, one can think about the following list, of which many techniques were briefly explained:

- Field Intensity Enhancement Factors
- Optical Reciprocity Theorem (ORT) and Plane Wave Excitation (PWE)
- Electrostatic Approximation (ESA)
- Image Dipole and Self-Reaction Field
- Generalized Mie Theory
- Spheroids and Prolate Spheroidal Coordinates: Helmoltz Equation
- E^4^ Approximation, used and presented in this article

Since complementary numerical analysis is desirable in order to simulate a device’s behaviors, the next section will focus on the Comsol Multi-Physics numerical method, which is used for simulations, and on its challenges.

### 4.2. Comsol Multi-Physics: Concerns and Considerations

Comsol Multi-Physics Software Package [28] is a numerical platform, based on the Finite Elements Method (FEM) [29,30]. The platform shares a large diversity of modules (i.e., specific domains) in Physics and Chemistry. Before reaching the step of the simulations themselves, there is a need to follow a step-by-step flow of design milestones. These milestones start with the design of the device’s geometry (shapes and polygones), the definition of the layers to be associated to these polygons, and the mesh definition of the elements (i.e., sub-distribution of small parts) on which the equations will apply. The mesh accuracy is crucial, since the accuracy of some critical zones depends on it. Well-experienced designers know how to deal with the trade-offs of such an optimization: Accuracy will always require longer run times, since the whole volume is divided into many more elements. This is why it is usually recommended to check the first runs with coarse FEM and then to gradually enhance the critical zones’ accuracy. Only then can functionality simulations and additional checks run. Sometimes, in order to simulate complex analyses, it is necessary to combine the usage of several additional modules. In addition to Comsol, and other kinds of Finite Elements Methods, it is sometimes necessary to use Matlab complementary software [31] for the mathematical modeling of some device behaviors.

### 4.3. Fabrication Methods

Successfully passing the design phase (geometry, layers, mesh, optimization), the analytical one (new models such as E^4^ approximation), and numerical ones (sanity checks and simulations runs), we now needed to choose and evaluate the best fabrication process for such SERS devices. At the end, prototypes composed of Silver (Ag) nanostructure arrays were fabricated, using Focused Ion Beam (FIB). However, as described here in detail, one can understand the concerns and considerations that rose all along the different steps of the fabrication process.

- **Sample Preparation**—Previous to the FIB steps, it was necessary to prepare the substrate to serve as an adequate sample. One initial suggestion was to use a dielectric substrate made of a glass microscope slide to be coated with silver using a vapor deposition process. Since such slides are not particularly smooth on the nanoscale, the question was raised regarding to what extent will this affect the smoothness of the surface of the silver layer and hence the variability in the height of the nanostructures. This is why the alternative of using quality silicon wafer was necessary. Moreover, the cleaning procedure of silicon is much easier and standard than that of glass.
- **FIB Accuracy**—Two types of equipment were available: One FIB, using a Gallium (Ga) beam, shares a resolution down to 13 nm, but in practice, it is more recommended for structures and separations above approximately 100 nm. Another FIB, using a Helium (He) beam, shares lighter particles; hence, it is easier to focus, and a greater resolution is available. Looking for high resolution, the He FIB was preferable; however, looking at the trade-off of the longer run time, Ga FIB was chosen. In order to assure a smooth move from design to FIB fabrication, STL files, which are fully compatible with the FIB operating software, were prepared with the layout of the pixels. In such a way, the design accuracy was respected.
- **Pixel Separation**—In order to resolve the signal from separate signals using a Raman microscope—remote imaging using a lens—the pixels must be separated by about 6 to 10 microns. For later designs using near field collection, this may not be necessary, which could be important, as discussed in the next item.
- **Large-Scale Lithography**—In addition to the resolution, the current design presents another challenge to FIB design: pixel separation. Since a flat metal sheet also provides Raman enhancement, the region between pixels could cause crosstalk. Thus, the metal layer in this ‘no-man’s land’ ought to be removed. Milling several microns width of the silver down to the dielectric substrate would likely prove-time consuming for the FIB, especially if several pixels are concerned.
- **Lithography**—Looking at the long-term industrial processing of several hundred pixels to be easily produced this way, the lithography process (masks, etching, etc…) was also considered. However, there is some understanding that rounded and pointed structures are an issue for the layer-by-layer methodology underlying lithography.
- **Surface Functionalization**—Following fabrication, a chemical process of surface functionalization is necessary. Surface activation is usually necessary to purify the surface of contaminants. However, surface activation is not necessary after vapor deposition and FIB milling if the specimen is immediately exposed to the functionalization treatment. Regarding the process itself, an overnight soak may be sufficient, but it may be more involved. The possibility of applying a Longmuir–Blodgett technique: the pMBA floats on the surface of a solution, after which the specimen is dipped vertically and methodically extracted. Surface tension causes the adhesion of a monolayer.
- **Optimal Aspect Ratio**—Homellhoff’s article [32] mentions that for an ellipsoidal nanostructure, for any given wavelength, there is an optimal aspect ratio, and not just the other way around (a resonant wavelength for a given aspect ratio). Thus, in order get the best performance from the design that is submitted to the FIB, it would be helpful to decide in advance what wavelength will be used.
- **Executive Summary**—The Ga FIB is the appropriate machine. Inserting the dielectric function of silver will give the optimal aspect ratio. Doing the same for gold and comparing the predicted enhancement will help decide which material to use. Using the results to create new STL or BMP files, which are compatible formats for the FIB processing, will enable a smooth move from design to fabrication. The simulations included arrays of nanocavities (Figure 14). The whole fabrication process ensured maximally sterile conditions.

## 5. Experimental Results

With a SERS pixel array that is capable of providing a spatially resolved measurement of chemical pH in a fluid, the ultimate goal is to provide real-time monitoring of a chemical reaction. To implement such a purpose, the pixels consist of a nanostructured substrate composed of an array of projections or cavities. Several parameters should be optimized. Among others, one can list the shape of the nanostructures, the thickness of the metallic (Ag or Au) layer to be tuned in order to give maximal enhancement at the desired wavelength, and the number and arrangement of nanostructures in order to obtain maximal responsivity.

### 5.1. Simulation Results: Nanocones, Nanoholes, and SERS Pixels Full Array

As presented in summary Table 2, the pixel dimensions of the total array active area (white space) are width × height = 1300 nm × 1080 nm, while the active area consists of an arrangement of 11 × 11 nanostructures. In a first try, the structures are depressions (open cavities) to be drilled into the silver layer. The opening is circular with a radius r = 20 nm. The separation between the structure centers is 120 nm, so the separation between the structure edges is 80 nm. Regarding the pixel depth and repetition, the following dimensions were chosen: 10 pixels separated by at least 5 to 10 µm for a good separation in an optical microscope. The structure of the first pixel should have a depth of 20 nm; i.e., it should be semi-spherical. The other pixels should be of increasing depth until a maximum depth of 120 nm. Since the aim was to determine plasmonic properties as a function of the ion dose, thus, it was important to record the current (amperage) and time used for each pattern. The following are the pictures of the array design and results. The architecture and design steps required a lot of optimization work until reaching the final array. Moreover, as presented in a previous publication [33], a lot of work focused on the Tip-Enhanced Raman Scattering (TERS) in order to define the optimal material and geometry of the individual tip-probe of the pixels array: hemisphere, cavity, hemispheroid, or nanocone.

### 5.2. Fabrication and Structural Charcaterization Results

**Figure 15:**
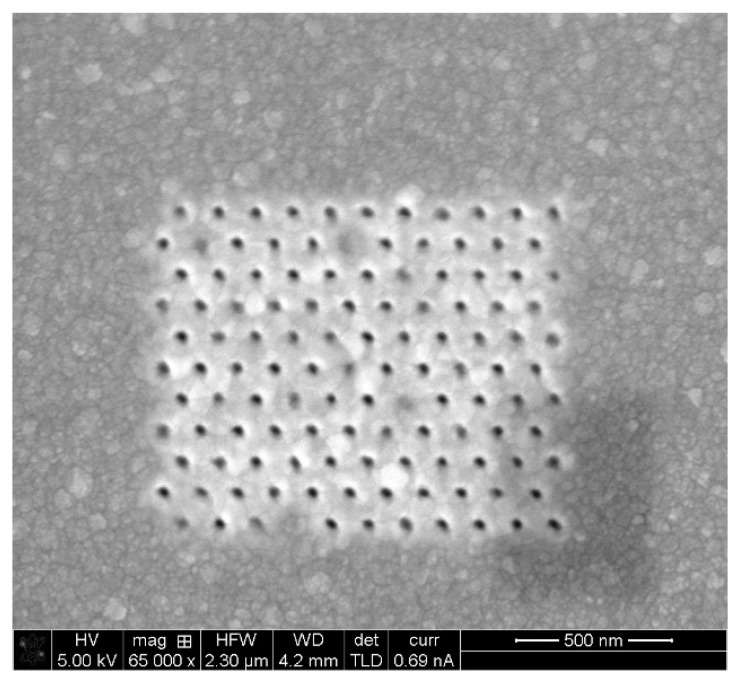
Pixel lattice fabrication using FIB. Dwell Time: 1.5 µs.

**Figure 16:**
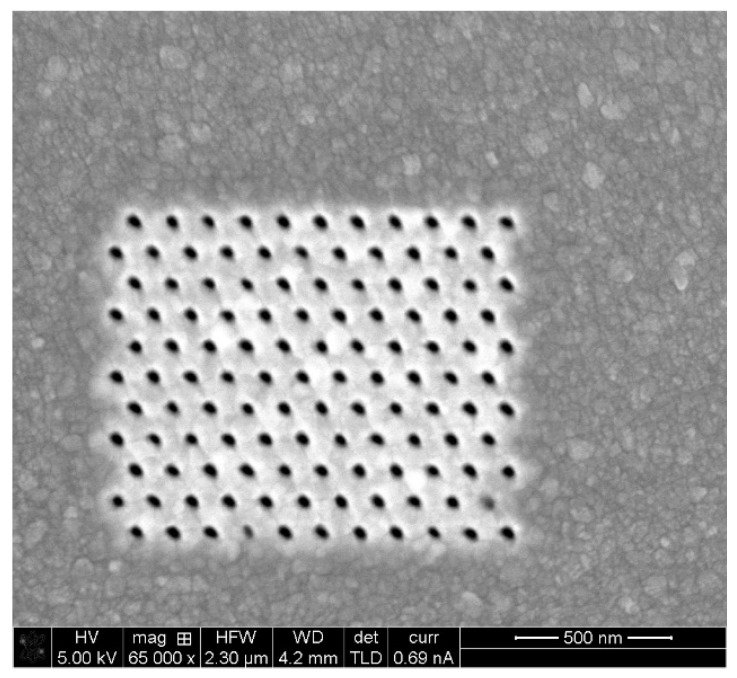
Pixel lattice fabrication using FIB. Dwell Time: 1.5 µs.

**Figure 17:**
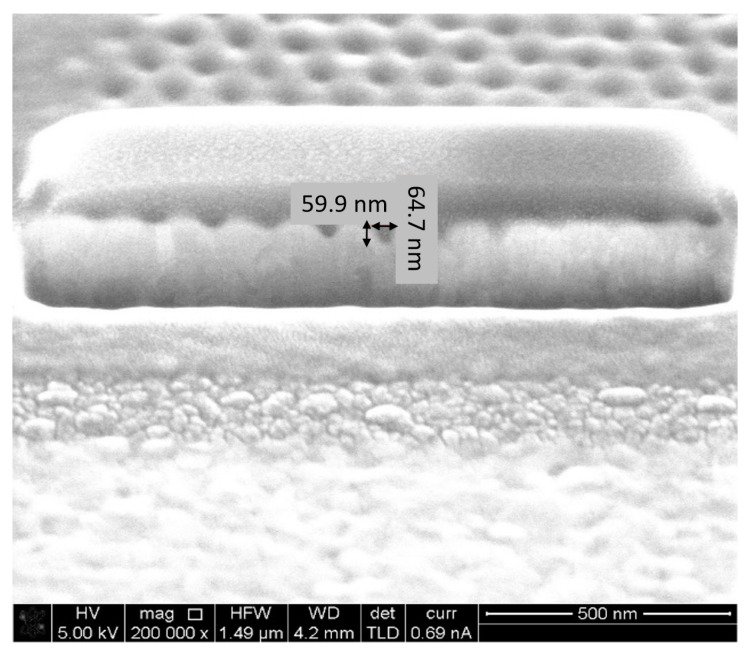
Nanohole array fabrication using FIB. Dwell Time: 1.5 µs; Depth: 65 nm.

**Figure 18:**
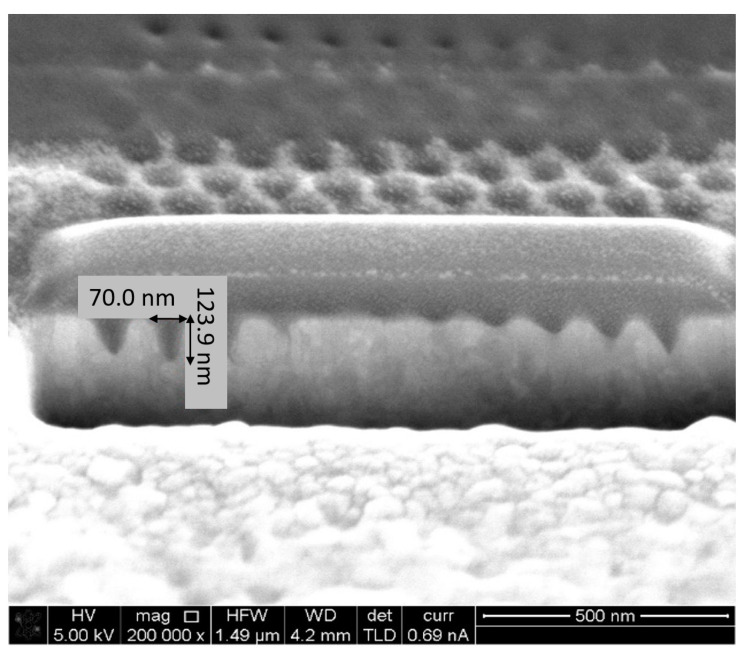
Nanohole array fabrication using FIB. Dwell Time: 1.5 µs; Depth: 124 nm.

The devices have been manufactured at Bar Ilan University’s Institute for Nanotechnology and Advanced Materials (BINA) using Focused Ion Beam (FIB) milling. Analytical instruments such as TEM and SEM were used to characterize the fabrication and monitor the quality. Several devices were fabricated using a variety of nanostructure configurations, specifically, square and hexagonal lattices. One device without nanostructures was also manufactured as control for the optical enhancement. In addition, the device was manufactured in two generations. In the first-generation device (GEN1), detection took place in the far field by sampling the scattered radiation through an appropriately located optic window. The second generation device (GEN2) employed near-field detection in which scattered radiation was received by a plasmonic antenna [34] located near the enhancement nanostructure, which transformed it to a propagating surface plasmon (PSPP) and thence to a propagating surface wave. The latter couples to a fiber optic waveguide allowing out-coupling and external detection. Figure 15, Figure 16, Figure 17 and Figure 18 present FIB-SEM results of the array and cavities. The pixel dimensions of the total array active area (white space) are width x height = 1300 nm × 1080 nm, while the active area consists of an arrangement of 11 × 11 nanostructures. In a first try, the structures are depressions (open cavities) to be drilled into the silver layer. The opening is circular with a radius r = 20 nm. The separation between structure centers is 120 nm, so the separation between structure edges is 80 nm. Regarding the pixel depth and repetition, the following dimensions were chosen: 10 pixels separated by at least 5 µm to 10 µm for a good separation in an optical microscope. The structure of the first pixel should have a depth of 20 nm, i.e., it should be semi-spherical. The other pixels should be of increasing depth until a maximum depth of 120 nm. Since the aim was to determine plasmonic properties as a function of the ion dose, thus, it was important to record the current (amperage) and time used for each pattern.

### 5.3. Techniques Survey of Optical Measurements, Detection, and Collection

**Figure 19:**
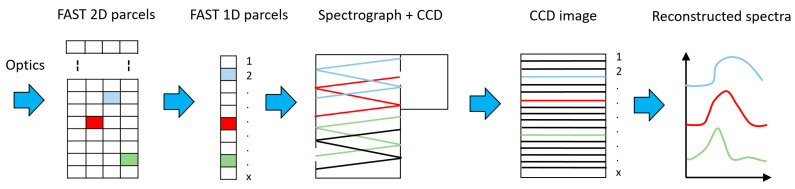
Fiber Array Spectral Translation (FAST) transformation main steps from 2D distribution to reconstructed spectra.

As pointed out in the Introduction, Raman imaging can be achieved by ‘classical’ methods including point scanning, line scanning, and wide field mapping. Newer methods that give instantaneous hyperspectral imaging include Fiber Array Spectral Translation (FAST), as presented in Figure 19, and Liquid Crystal Tunable Filter (LCTF) [9]. Thus, the latter are to be preferred. In particular, in a FAST-based design, the emitted Raman radiation is imaged by an optical objective onto an array of optic fibers (FAST), each pixel onto one fiber (at least). These emit the collected light through a dispersive element (such as a grating), separating it into its component spectra onto an array of photodiodes, gated detectors, or onto a CCD, one row per fiber. Thus, the Raman spectrum of each pixel is imaged simultaneously. In later stages of the first-generation device, the aim is to replace the lens-based imaging—with its inherently larger distances—with a direct collection by a proximally located Fiber Array. Communication lines will include two arrays of waveguides for delivery and collection of the light to each one of the pixels in the array. The challenge will be to design a grid of baffles to prevent *crosstalk*—an unwanted collection of radiation from neighboring pixels. Plasmonic nanoantennae coupled to plasmonic waveguides for collection and excitation will be considered as a more compact alternative for second-generation design.

### 5.4. Preferred Technique: Saddle Point Integration Method for Spatial Coordinate Transformation

**Figure 20:**
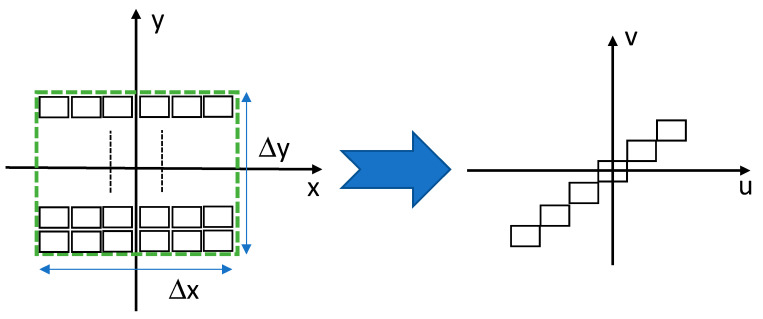
Coordinates transformation from 2D distribution to 1D diagonal distribution.

At the end, the optimal solution is the following, which we propose. The aim is to perform a 2D to 1D spatial conversion. We intend to do this coordinate transformation using the method of saddle point integration [35]. We assume that the information distribution is within the rectangular region of interest as described in the left side of Figure 20 and that the 2D information is about to be transformed into a 1D diagonal vector of distribution. We assume that the original resolution along the vertical axis is δy, and along the horizontal axis x, it is δx.

**Figure 21:**
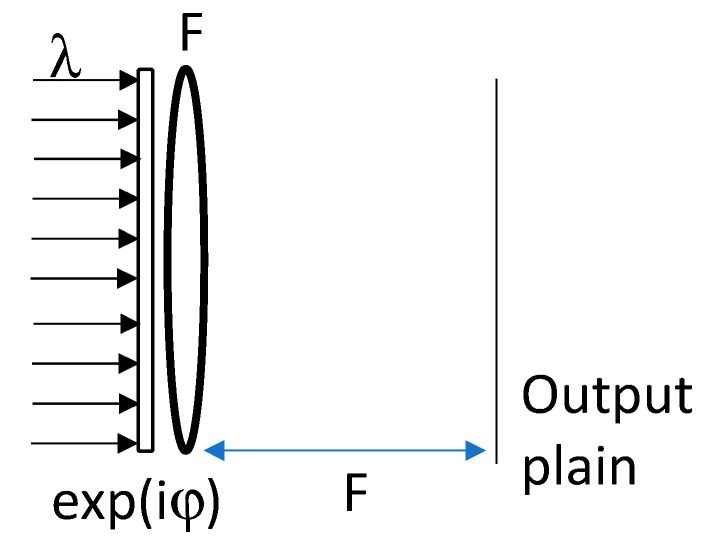
The coordinate transformation optical setup.

According to the equations of saddle point integration, we aim to design a phase-only element that has a phase of φ(x,y), and when the positioned is attached to an optical lens with a focal length of F and illuminated with a wavelength of λ, it performs the desired 2D to 1D coordinate transformation at the output plain of Figure 21.

The equations that need to be fulfilled are as follows:

(14) $$\frac{\partial \varphi }{\partial x}=\frac{2\pi u}{\lambda F}\;\;\;\;\;\;\;\;\frac{\partial \varphi }{\partial y}=\frac{2\pi v}{\lambda F}$$

Meanwhile, we aim to have the 2D to 1D coordinate transformation which aims to:

(15) $$u\left(x,y\right)=\left(x+\frac{\Delta x}{2}\right)+\frac{\left(y+\frac{\Delta y}{2}\right)}{\delta y}\Delta x=\frac{\Delta x}{2}+\frac{\Delta x\Delta y}{2\;\delta y}+x+\frac{\Delta x}{\delta y}y$$

(16) $$v\left(x,y\right)=\frac{\Delta x}{y}u\left(x,y\right).$$

Substituting Equation (15) into the differential Equation (14) gives the following solution for the phase of the phase-only element that will perform the required coordinate transformation:

(17) $$\varphi \left(x,y\right)\;=\;\frac{\Delta x}{\delta y}\frac{2\pi }{\lambda F}\left(\frac{\Delta x}{2}\;+\;\frac{\Delta x\Delta y}{2\;\delta y}\right)y\;+\;\frac{\pi }{\lambda F}{\left(\frac{\Delta x}{\delta y}\right)}^{2}y^{2}\;+\;\frac{2\pi }{\lambda F}\frac{\Delta x}{\delta y}xy\;+\;\frac{\pi }{\lambda F}x^{2}\;\;+\;\frac{2\pi }{\lambda F}\left(\frac{\Delta x}{2}\;+\;\frac{\Delta x\Delta y}{2\;\delta y}\right)x$$

## 6. Conclusions

This article reported the design, formalism, simulations, and construction of a device for the spatial mapping of chemical pH in a solution undergoing a chemical reaction. The device utilizes the methods of SERS for measuring the pH. Central to the proposed device is a regular two-dimensional arrangement of so-called pixels, with an arrangement of 11 × 11 pixels. Pixels were constructed employing a design consisting of a cluster of metallic nanoscale structures on a dielectric substrate. Numerical PDE solvers and optimizers were employed to optimize the Raman enhancement as a function of nanostructure shapes, dimensions, spacing distances, and cluster arrangements, as well as parameters of the excitation radiation. Following this numerical guidance, the structures were fabricated by FIB on a silver layer sputtered on a silicon wafer, which displays minimal absorption in the range of its LSP resonant frequency. The nanostructure displays a base radius of 20 nm and an aspect ratio of 3, corresponding to a high eccentricity value close to 0.95 with a minimal radius of curvature of about 7 nm at the tip. The structures were arranged with a center-to-center separation of 75 nm—a separation of 35 nm between structure surfaces—giving a total cluster dimension of 0.75 µm on each side. The total pixel dimension is less than 2 µm.

Looking forward to the next steps, and after fine-tuning of the fabrication process, several milestones will be achieved. Among others, the plasmon scattering spectrum, Raman scattering, and pH measurements will be performed. In parallel to the measurements, a series of complementary and complex optical simulations will complete the analysis.

## Figures and Tables

**Table 1:** Enhancement Quantities for Particles and Cavities.

Symbol	Particle		Cavity	
$M_{\max}\left(\lambda \right)$	${\left|\frac{\epsilon \left(\lambda \right)}{L_{3}\epsilon \left(\lambda \right)+\left(1-L_{3}\right)\epsilon_{M}}\right|}^{2}$	(2)	$\tilde{M}={\left|\frac{\epsilon \left(\lambda \right)}{\left(1-L_{3}\right)\epsilon \left(\lambda \right)+L_{3}\epsilon_{M}}\right|}^{2}$	(3)
$M_{\min}=M_{\mathrm{in}}$	${\left|\frac{\epsilon_{M}}{L_{3}\epsilon \left(\lambda \right)+\left(1-L_{3}\right)\epsilon_{M}}\right|}^{2}$	(4)	N/A	
$\epsilon_{\mathrm{re}}\left(\lambda_{*}\right)\;\;$	$-\frac{1-L_{3}}{L_{3}}\epsilon_{M}$	(5)	$-\frac{L_{3}}{1-L_{3}}\epsilon_{M}$	(6)
$M_{\max}\left(\lambda_{*}\right)$	9(3L3)2(1−L3L3)2|ϵMImϵ(λ*)|2	(7)	9(3−3L3)2(L31−L3)2|ϵMImϵ(λ*)|2	(8)

**Table 2:** Nanostructure dimensions of the SERS array.

Nanostructure Geometry	Prolate Spheroid
Material	Ag
Eccentricity	0.943
Aspect ratio	3.00
Base radius	20 nm
Separation	120 nm
Array size	1300 nm × 1080 nm
Array area	1.40 µm^2^
Array size (Number of structures)	11 × 11
Total structures	121
